# Simultaneous Detection of SARS-CoV-2 and Influenza Virus in Wastewater of Two Cities in Southeastern Germany, January to May 2022

**DOI:** 10.3390/ijerph192013374

**Published:** 2022-10-17

**Authors:** Roger Dumke, Michael Geissler, Annett Skupin, Björn Helm, Robin Mayer, Sara Schubert, Reinhard Oertel, Bertold Renner, Alexander H. Dalpke

**Affiliations:** 1 Institute of Medical Microbiology und Virology, University Hospital Carl Gustav Carus, Technische Universität Dresden, 01307 Dresden, Germany; 2 Institute of Urban and Industrial Water Management, Technische Universität Dresden, 01069 Dresden, Germany; 3 Institute of Clinical Pharmacology, Faculty of Medicine Carl Gustav Carus, Technische Universität Dresden, 01307 Dresden, Germany; 4 Institute of Hydrobiology, Technische Universität Dresden, 01217 Dresden, Germany; 5 Department of Infectious Diseases, Medical Microbiology and Hygiene, University Hospital Heidelberg, 69120 Heidelberg, Germany

**Keywords:** influenza virus, SARS-CoV-2, molecular detection, wastewater-based epidemiology

## Abstract

Dependent on the excretion pattern, wastewater monitoring of viruses can be a valuable approach to characterizing their circulation in the human population. Using polyethylene glycol precipitation and reverse transcription-quantitative PCR, the occurrence of RNA of SARS-CoV-2 and influenza viruses A/B in the raw wastewater of two treatment plants in Germany between January and May 2022 was investigated. Due to the relatively high incidence in both exposal areas (plant 1 and plant 2), SARS-CoV-2-specific RNA was determined in all 273 composite samples analyzed (concentration of E gene: 1.3 × 10^4^ to 3.2 × 10^6^ gc/L). Despite a nation-wide low number of confirmed infections, influenza virus A was demonstrated in 5.2% (concentration: 9.8 × 10^2^ to 8.4 × 10^4^ gc/L; plant 1) and in 41.6% (3.6 × 10^3^ to 3.0 × 10^5^ gc/L; plant 2) of samples. Influenza virus B was detected in 36.0% (7.2 × 10^2^ to 8.5 × 10^6^ gc/L; plant 1) and 57.7% (9.6 × 10^3^ to 2.1 × 10^7^ gc/L; plant 2) of wastewater samples. The results of the study demonstrate the frequent detection of two primary respiratory viruses in wastewater and offer the possibility to track the epidemiology of influenza by wastewater-based monitoring.

## 1. Introduction

Municipal wastewater represents a reservoir of numerous pathogens entering the water cycle after excretion with the feces of infected persons. In consequence, concentration of different parasites, bacteria, or viruses in wastewater can be used to investigate their circulation in the population of the catchment area of a wastewater treatment plant (WWTP), thus serving as a population-based surveillance system independent of patient testing strategies. Using this approach, so-called wastewater-based epidemiology (WBE) is an interesting additional tool for characterizing the epidemiological situation regarding distinct infectious diseases. Previously, “virological” WBE was used to confirm the success of immunization programs for poliovirus eradication or to track qualitative and quantitative trends of occurrence of the classical viruses causing diarrhea, such as norovirus or rotavirus [1]. Recently, analysis of wastewater in many countries was performed to monitor the prevalence of SARS-CoV-2 [2,3]. Despite the fact that infections with coronaviruses result mainly in respiratory tract symptoms of patients, the frequent emergence of specific RNA in stool samples [4,5,6,7] allows a reliable estimation of the course of incidences in the population connected to treatment plants. Besides a relatively low cost, further advantages of this approach are its independence from the symptomatic or asymptomatic status of infected persons, as well as from the extent and methodology of patient testing procedures. On the other hand, many factors influence the significance of wastewater monitoring data, such as methodic aspects of virus detection (efficiency of virus enrichment, RNA/DNA preparation, primer/probe combination for detection, and standard used for quantification), presence of PCR inhibitors, specific local features (composition of wastewater, construction of the sewer system), demographic and regional characteristics of the served population, as well as changes of viruses of interest (e.g., emergence of variants with differing excretion profile), respectively.

In principle, WBE offers the possibility to include further pathogens with properties initiating an epidemic or even a pandemic situation. Influenza virus A is especially well-known for its potential to cause worldwide epidemics with severe cases of infection [8]. Similar to SARS-CoV-2, influenza viruses are frequently excreted by the feces of patients with confirmed infection [9,10], representing the prerequisite for the wastewater monitoring of the pathogen. However, the number of studies investigating the occurrence of influenza virus in municipal wastewater so far is limited. Whereas the presence of viruses in the environment surrounding outbreak areas was described [11,12,13,14], only two reports to date characterized the occurrence of viruses in Dutch and Polish sewage influents, and finding influenza virus A in 1 of 10 (2.6 × 10^5^ genome copies (gc)/L) and in 1 of 15 samples (1.3 × 10^5^ gc/L), respectively [15,16]. Further investigations are urgently needed to evaluate the value of WBE for the characterization of future influenza outbreaks. This could be helpful for complementing other surveillance systems for influenza that are run in different countries, and from which results are used to define the influenza season, as well as to delineate measures of infection control. It is important to note that, in time periods with administrative restrictions to reduce SARS-CoV-2 infections, studies from Germany and other countries reported a strong decrease in the number of confirmed influenza cases [17,18,19,20,21]. However, it remains to investigate whether this low level of clinical positivity will be reflected in data from municipal wastewater.

Here, the first results of the detection of influenza virus A/B and SARS-CoV-2 in the influent of two WWTPs in Germany are presented. As these viruses are transmitted by the same main epidemiological route (via aerosols after close person-to-person contact) and SARS-CoV-2 infections continued in the population throughout the sampling period, concentrations of both viral parameters were compared to obtain further insights into their parallel occurrence in raw wastewater.

## 2. Materials and Methods

Between 10 January and 29 May 2022, two WWTPs in southeastern Germany (in the federal state Saxony) were sampled daily. In all cases, 24-h composite samples were used for virological investigations. The WWTPs treat the wastewater of 251.000 (plant 1) and 702.000 inhabitants (plant 2). The ratios of combined/separate sewers are 62/38% and 75/25%, respectively. Raw wastewater samples were refrigerated (4 °C) and processed within 3 days. After centrifugation to remove particulate matter (4 °C, 3300× *g*, 25 min), supernatants (45 mL) were concentrated by polyethylene glycol (PEG) precipitation, as described in [22]. Briefly, after the addition of NaCl and PEG 8000 (Roth, Karlsruhe, Germany), the suspension was mixed overhead (room temperature, 25 min) and ultracentrifuged (12,000× *g*, 1.5 h, 4 °C). Pellets were resuspended in phosphate buffered saline (pH 7.4; Gibco, Paisley, UK), resulting in volumes of concentrates between 400 and 600 µL.

The RNA from 200 µL of concentrate was prepared using RNeasy columns (Qiagen, Hilden, Germany) according to the manufacturer’s recommendation and was additionally treated to remove PCR inhibitors (Zymo Research, Irvine, CA, USA). The same nucleic acid isolation was used for the detection of crAssphage as a surrogate virus for the human fecal pollution of water. The comparison of RNeasy column eluates with DNA from the same wastewater concentrates prepared with the QIAamp DNA mini kit (Qiagen, Hilden, Germany) demonstrated similar crossing thresholds (Ct; data not shown). The processing of wastewater samples was strictly separated from the analysis of viruses in the clinical samples. Additionally, RNA extraction and RT-qPCR setup were performed in different laboratories.

Detection of viruses by reverse transcription quantitative real-time-PCR (RT-qPCR) was performed with commercial kits (Altona Diagnostics, Hamburg, Germany), amplifying the E and S gene of SARS-CoV-2 (RealStar SARS-CoV-2 RT-PCR kit 1.0), as well as the matrix protein gene of influenza virus A and B (RealStar Influenza Screen & Type RT-PCR Kit 4.0). Samples were tested in duplicate in a QuantStudio 5 cycler (Thermo Fisher Scientific, Waltham, MA, USA) with positive (included in the kit), negative (water), and extraction controls (internal control) in each run. Regarding SARS-CoV-2, only test results which gave signals after the amplification of both genes, and as for influenza viruses, Ct values ≤ 40, were considered as positive. For the straightforward presentation of the results of SARS-CoV-2 testing, Ct values of the E gene were used. Calculation of the virus concentrations was carried out by using standard curves of RNA standards of SARS-CoV-2, Wuhan strain (Twist Bioscience, San Francisco, CA, USA; [22]) and of influenza virus A/B (AccuPlex Flu A/B and RSV verification kit; LGC Seracare, Milford, MA, USA). To confirm real-time-PCR-positive influenza virus A/B detections and to further characterize the pathogen(s) in randomly selected RT-qPCR-positive samples, nested PCR was performed according to standard procedures and published protocols [23]. Primers were designed after the alignment of the sequences of influenza virus A/B in the GenBank database and are summarized in Table S1. Reverse transcription-PCR was performed by using the SS III one-step RT-PCR Platinum Taq HiFi kit (Invitrogen, Waltham, MA, USA; influenza virus A) and the OneStep RT-PCR kit (Qiagen; influenza virus B). Agarose gel checked amplification products were treated with MSB Spin PCRapace columns (Invitek, Berlin, Germany) according to the instructions of the manufacturer and Sanger sequenced bi-directionally (SeqLab, Göttingen, Germany). The results were compared with sequences in the NCBI GenBank database using BLAST. Detection of crAssphage was carried out in a CFX Opus 96 thermocycler (Bio-Rad, Hercules, CA, USA), as described in [24]. For amplification, a CPQ_064 primer/probe combination and a HotStart Taq master mix kit (Qiagen) were used. The data of the SARS-CoV-2 detection were adjusted with the measured concentrations of crAssphage, as described recently [25]. To convert Ct values into concentrations of genome copies, an amplification product with a confirmed crAssphage sequence was cloned into the pCR2.1-TOPO vector (Invitrogen), which was used for transformation of the TOP10F’ cells (Thermo Fisher), as described by the manufacturer. The plasmid of a propagated strain carrying the crAssphage-specific insert was isolated with the QIAprep Spin Miniprep Kit (Qiagen), photometrically quantified. and tenfold dilutions of plasmid were investigated to establish the standard curve.

## 3. Results

**Figure 1:**
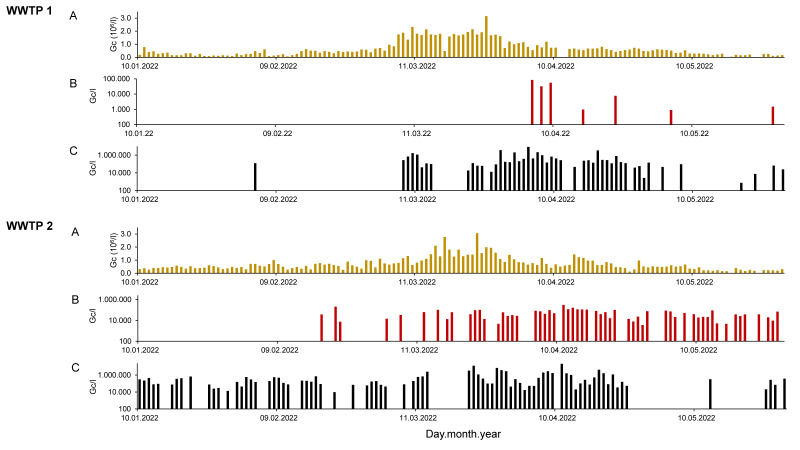
Concentrations of genome copies (gc) of SARS-CoV-2 (E gene; (**A**)), influenza virus A (**B**) and B (**C**) in raw wastewater of WWTPs 1 and 2. Bars represent mean gc/L of two technical RT-qPCR amplifications for each sample.

Overall, 136 (plant 1) and 137 (plant 2) wastewater samples were investigated. In all samples, SARS-CoV-2-specific RNA was determined, and E gene concentrations between 2.5 × 10^4^ and 3.2 × 10^6^ gc/L (mean: 6.6 × 10^5^ gc/L; plant 1) and 1.3 × 10^4^ and 3.1 × 10^6^ gc/L (mean: 7.0 × 10^5^ gc/L; plant 2) were calculated (Figure 1). As expected, not only the genome concentrations of SARS-CoV-2 were comparable between the nearly located WWTPs, but also the time courses of the viral load in the wastewater of both cities were similar. The highest concentrations of SARS-CoV-2 genome copies were detected during March 2022 (with an increasing presence of Omicron variants BA.4 and BA.5 in the populations of both cities; https://www.coronavirus.sachsen.de/infektionsfaelle-in-sachsen-4151.html#a-10365, accessed on 28 June 2022). In general, SARS-CoV-2 concentrations follow the reported incidence in the population of the catchment areas of the plants (Figure S1). To assess the influence of extraordinary events (e.g., heavy rainfall) on data, concentrations of the ubiquitous crAssphage were determined and used to adjust the values of the E gene of SARS-CoV-2 (Figure S2). Mean quotients of crAssphage and E gene genome concentrations of 0.10 ± 0.07 (min.: 0.01/max.: 0.32) in plant 1 samples and 0.06 ± 0.04 (min.: 0.01/max.: 0.24) in plant 2 samples were calculated, indicating that factors of strong importance for SARS-CoV-2 and influenza virus concentrations in wastewater could not be identified by this approach.

Influenza virus A was demonstrated in 5.2% (*n* = 7) of the samples from treatment plant 1 and in 41.6% (*n* = 57) of the samples from plant 2. According to the standard curves (Figure S3), concentrations between 9.8 × 10^2^ and 8.4 × 10^4^ gc/L (plant 1) and 3.6 × 10^3^ and 3.0 × 10^5^ gc/L (plant 2) were calculated. In contrast, influenza virus B was detected in 36.0% (*n* = 49; plant 1) and in 57.7% (*n* = 79; plant 2) of the sampled wastewater. Concentrations ranged between 7.2 × 10^2^ and 8.5 × 10^6^ gc/L (plant 1) and 9.6 × 10^3^ and 2.1 × 10^7^ gc/L (plant 2). Using a Ct value of 40, detection limits of 360 gc/L (influenza virus A) and 170 gc/L (influenza virus B) can be estimated. Both influenza virus A and B were found in 14.3% of samples (plant 1: 3.7%, plant 2: 24.8%).

Results of the Sanger sequencing of the amplification products of conventional PCR of both influenza virus A- and B-positive samples confirmed the specificity of the RT-qPCR approach used: in five samples which were positive for influenza virus A, a 100% identification with the segment 7 matrix protein 2 (M2) and matrix protein 1 (M1) sequences of different influenza virus A strains (H3N2) deposited in GenBank was found. In 20 samples positive for influenza virus B after RT-qPCR, the identity of the resulted sequences with the segment 7 matrix protein 1 (M1) and BM2 protein (BM2) genes of influenza virus B was confirmed. Furthermore, missing amplification products after the analysis of 20 RT-qPCR-negative wastewater concentrates verified the sensitivity of the influenza virus detection method.

## 4. Discussion

In this study, the presence of the RNA of respiratory viruses in the municipal wastewater of two cities in Germany was demonstrated. Despite a similar transmission route in the human population and the comparable influence of inactivation factors in the environment (e.g., temperature), the data indicate that SARS-CoV-2 and influenza virus do not peak at the same time points. Influenza virus A detection occurred after April 2022 in only 5% of the samples from plant 1, but it was found regularly after the end of February in plant 2. The RNA of influenza virus B could be demonstrated with a high frequency of 36% and 58% in both plants. In contrast, concentrations of SARS-CoV-2 follow the emergence and spread of variants with a higher potential of transmission (Omicron). Additionally, public health measures to reduce the rate of respiratory infections (lockdowns, mandatory mask-wearing) influence the time-dependent presence of specific RNA of viruses in wastewater. With the relatively high incidence of infections with SARS-CoV-2 in the catchment areas (Figure S1) during the study period (plant 1: 94 to 2.637 and plant 2: 120 to 1.997/100.000 inhabitants), the precondition for the constant RNA positivity of all investigated wastewater samples was given. It is important to note that this study was carried out over a limited period of time and in two cities only. Long-term and nation-wide investigations are necessary to understand temporal and regional patterns of the co-occurrence of respiratory viruses in wastewater.

Besides other factors, the efficiency of the method used for the concentration of viruses in wastewater is important for the results of the water monitoring of viruses. PEG precipitation has been used in many investigations to analyze SARS-CoV-2 in wastewater [26]. Recently, a recovery rate of SARS-CoV-2 after PEG precipitation of 59% was demonstrated, which was superior to that obtained via Vivaspin and Centricon ultrafiltration columns ([22]; data not shown). This report demonstrates the co-detection by RT-qPCR of two enveloped viruses in wastewater from concentrates after PEG precipitation. It is a limitation of the study that quantitative results about the recovery of influenza virus A and B by precipitation are missing. However, the data confirm that the enrichment of raw wastewater by this simple concentration procedure results in the frequent detection of SARS-CoV-2 and influenza virus A/B. Thus, wastewater surveillance of both pathogens using PEG-precipitated samples might minimize the technical effort in the laboratory and the costs for different virus concentration procedures.

Here, it proved to be difficult to confirm the results of positive RT-qPCR by sequencing. Results of the direct sequencing of the products of RT-qPCR could not be evaluated. After the re-amplification of wastewater concentrates that tested positive by RT-qPCR with nested PCR, clear bands were obtained for influenza virus A. Unfortunately, and due to the limited volume, only some RNA samples were available for investigation with this approach. Furthermore, nested PCR for the sequencing of influenza virus B resulted in multiple bands without a signal at the expected 209/302 bp (Table S1). In comparison to clinical materials, an influence of inhibitory substances in concentrated wastewater samples on RT-PCR cannot be excluded. After the re-amplification of the influenza virus B-positive RT-qPCR products with the primer pair InflA/Bf and InflA/Br (Table S1), relatively short sequences (about 40 bases) could be successfully used for comparison with sequences deposited in GenBank. Unfortunately, with this approach, it is not possible to differentiate the two lineages of influenza virus B. Future studies should optimize the procedure to characterize the types of influenza viruses occurring in wastewater. Using this method in addition to the typing of strains in clinical samples, our knowledge about strain circulation will be deepened [27], and an extended interpretation of changes in the presence of lineages [28] can be performed. Especially for influenza virus A, parallel occurrence of different types in a given sample cannot be excluded and was demonstrated in a previous study [15]. Furthermore, co-circulation of influenza virus A and B in the human population was described [29,30] and can consequently be expected in the wastewater. Using cloning and next-generation sequencing approaches, the extent of the variability of influenza viruses in wastewater should be analyzed in future investigations. This approach might be helpful for the epidemiological evaluation of virus circulation in human populations, as well as in agricultural wastewater (e.g., poultry farms).

Based on information from the operators of both plants, a relevant influence of agricultural wastewater on inflow is not probable. In consequence, a predominantly human origin of measured influenza A and B must be assumed. Further sources (wild birds, waste from poultry farms) can be important for distinct WWTPs and were discussed as causes of the frequent detection (40%) of influenza virus A in Dutch rivers [15]. Parallel investigation of crAssphage gave no hint that meteorological or other events had a strong influence on the concentrations of SARS-CoV-2 and influenza virus A/B. Meanwhile, detection of this ubiquitous phage is generally accepted as a marker for tracking the contamination of water with human feces [31] and has been used for the interpretation of the results of wastewater surveillance [32]. According to the very limited data available to date, the extent of entries by non-human and agricultural sources into surface waters cannot be evaluated and needs further investigation.

In the present study, the measured mean concentrations of influenza virus A in the wastewater of both cities are below the values (2.6 × 10^5^ gc/L and 1.3 × 10^5^ gc/L) obtained from Dutch and Polish WWTPs, which are available for a quantitative comparison [15,16]. Besides methodic aspects (differing virus concentration and amplification procedures, as well as standards used for quantification), data from The Netherlands came from the time period of the influenza virus A (H1N1) 2009 pandemic, which could result in a stronger input of these viruses into wastewater. In addition, local conditions, such as sewer design and hydraulic retention time in the sewer, will influence the decay rates of viruses in sewage. Especially in enveloped viruses, temperature, adsorption, and the activity of microorganisms are characterized as important factors of virus persistence in water [33,34], and the variability of these factors can be important for differences in the quantitative occurrence of viruses in influents of treatment plants. Future investigations under conditions of more pronounced influenza seasons will show which levels of virus concentrations can be expected in WWTPs. The reported cases of confirmed influenza infections in Saxony in the study period (until the 21st calendar week of 2022) reached 3.743 (https://rki.de, accessed on 7 July 2022), which is higher than in the comparable period of 2021 (*n* = 32) but much lower than in the years 2019 (20.266) and 2020 (22.495), respectively. During the study period, the weekly reports of the National Working Group for Influenza (https://influenza.rki.de, accessed on 7 July 2022) confirmed an increased detection of influenza virus A strains between the middle of April and the end of May 2022. Interestingly, data from the German reference center for influenza virus, which is based on samples from the national influenza sentinel network, showed that, up to the 21st calendar week of 2022, 205 influenza strains had been typed. Among them, 192 (93.6%) were classified as H3N2, 11 (5.4%) as H1N1pdm09, and only 2 (1.0%) as influenza B (https://influenza.rki.de, accessed on 7 July 2022). Whereas the sequencing results of influenza virus A-positive wastewater samples supported the dominance of the H3N2 type, the frequent detection of influenza virus B in the water of both cities is striking. Differences in the course of infections by influenza virus A and B [35], with the consequence of a possible underestimation of circulating B viruses among patients, might explain the high rate of positivity in municipal wastewater. In addition, in agreement with the epidemiology of SARS-CoV-2 infections, influenza virus A/B-infected but asymptomatic patients have been described [36]. Despite the fact that these persons might show a lower and shorter viral shedding period, a substantial contribution to the virus load in wastewater cannot be excluded and needs additional investigation.

## 5. Conclusions

The preliminary results of the present study confirm the frequent detection of the RNA of influenza viruses A and B in the raw wastewater of two cities in Germany. Further studies should include a greater number of samples from different regions to expand our knowledge about the occurrence of these viruses in water resources. Especially under conditions of missing restrictions to reduce the transmission of respiratory virus infections (a permanently low incidence of SARS-CoV-2 infections and/or a limited impact of severe cases of COVID-19 on the public health system) and due to the low incidence of Flu cases in the last seasons, an increased number of patients with confirmed influenza virus infection must be expected in the future. The data suggest that wastewater analysis might be a promising additional tool for characterizing the prevalence of influenza viruses in the catchment area of a WWTP. As established for later waves of SARS-CoV-2 pandemics, a timely and dense monitoring network for influenza virus detection in wastewater could not only help to evaluate the epidemiological situation in the populations served by WWTPs, but also possibly represents an early warning tool for starting counteractive measures, such as the enhancement of immunization programs.

## Data Availability

The data presented in this study are available upon request from the corresponding author.

## Supplementary Materials

The following supporting information can be downloaded at: https://www.mdpi.com/article/10.3390/ijerph192013374/s1, Figure S1: Concentration of SARS-CoV-2 in the wastewater and corresponding incidence of SARS-CoV-2 infections in the population served by WWTPs 1 and 2 (according to the data in the national database of the Robert-Koch-Institut, Berlin, Germany); Figure S2: Concentrations of SARS-CoV-2 (E gene) and crAssphage, and crAssphage-adjusted concentrations of SARS-CoV-2 (SARS-CoV-2 E gene gc/crAssphage gc) in wastewater of WWTPs 1 and 2; Figure S3: Standard curves of real-time-PCR for detection of influenza virus A and B (Influenza Screen & Type RT-PCR Kit 4.0; Altona Diagnostics) after testing 1:4 dilutions (5 parallels per dilution) of standard (AccuPlex Flu A/B and RSV verification kit; LGC Seracare); Table S1: Primer used for characterization of influenza virus A/B in real-time-PCR-positive wastewater samples.
