# Supplementary material for: Simultaneous Detection of SARS-CoV-2 and Influenza Virus in Wastewater of Two Cities in Southeastern Germany, January to May 2022
Source: Int J Environ Res Public Health. 2022 Oct 17;19(20):13374. doi: 10.3390/ijerph192013374 (PMC9603229; doi:10.3390/ijerph192013374)
Supplement: Supplementary file 1 [file ijerph-19-13374-s001.zip › ijerph-1906412-supplementary.pdf]

Table S1. Primer used for characterization of influenza virus A/B in real-time PCR-positive wastewater samples.

Virus	Gene	Name	Description	Sequence (5‘ - 3‘)	Product size	Source
Influenza A/B	Matrix protein	InflA/Bf	Re-amplification of product of RT qPCR	GGA GAA GGC AAA GCA GAA CTA G	107 bp	This study
		InflA/Br		GCA TCT TTT GTT TTT TAT CCA TTC		This study
Influenza A	Matrix protein	MF1MR1027f	First PCR	AGC AAA AGC AGG TAG ATA TTG AAA GA	1,027 bp	WHO (2021)
		MF1MR1027r	First/seminested PCR	AGT AGA AAC AAG GTA GTT TTT TAC TC		WHO (2021)
		MF1MR1027fn	Seminested PCR	GAG TCT TCT AAC CGA GGT CGA AAC	1,000 bp	This study
Influenza B						
Yamagata	Hemagglutinin	BYf226	First PCR	ACA CCT TCT GCG AAA GCT TCA	284 bp	WHO (2021)
		BYr613	First PCR	CAT AGA GGT TCT TCA TTT GGG TT		WHO (2021)
		BYf226n	Nested PCR	CTA TAA TGC ACG ACA GAA CAA	209 bp	This study
		BYr613n	Nested PCR	GTT ATC TGA ATG GAA CCC		This study
Victoria	Hemagglutinin	BVf224	First PCR	ACA TAC CCT CGG CAA GAG TTT C	388 bp	WHO (2021)
		BVr507	First PCR	TGC TGT TTT GTT GTT GTC GTT TT		WHO (2021)

	BVf224n	Nested PCR	CTG TTA CAT CTG GGT GCT TTC	302 bp	This study
	BVr507n	Nested PCR	CCA AGC CAT TGT TGC GAA AAA		This study

World Health Organization: WHO information for the molecular detection of influenza viruses. February 2021. https://cdn.who.int/media/docs/default-source/influenza/molecular-detention-of-influenza-viruses/protocols_influenza_virus_detection_feb_2021.pdf?sfvrsn=df7d268a_5.

WWTP 1

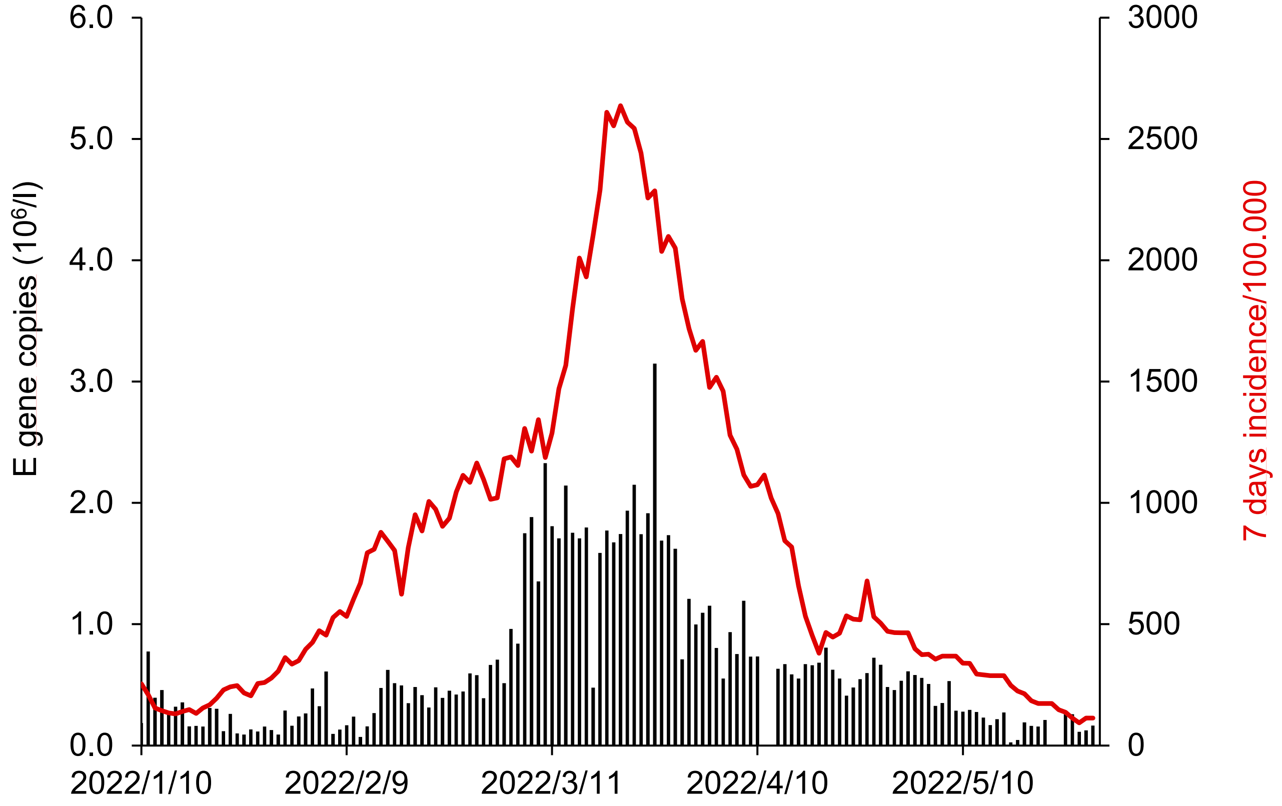

WWTP 2

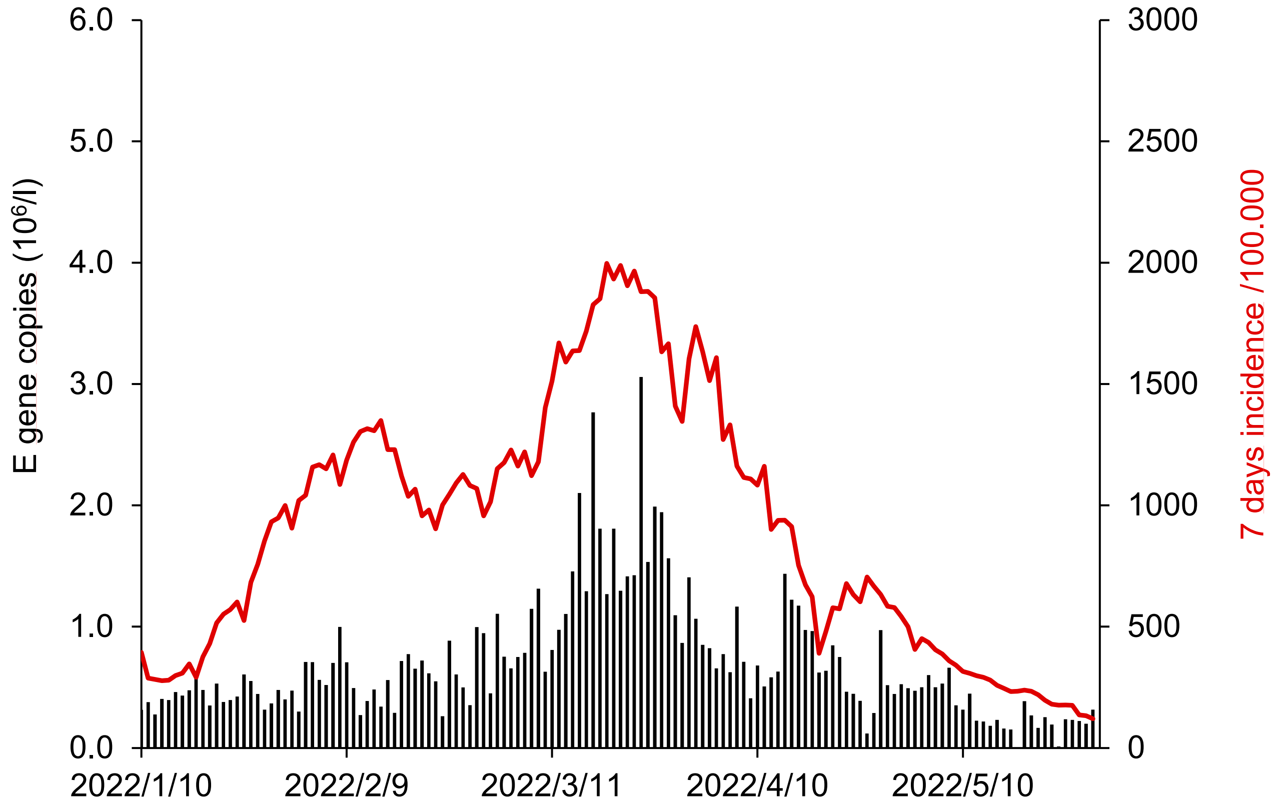

Figure S1. Concentration of SARS-CoV-2 in the wastewater and corresponding incidence of SARS-CoV-2 infections in the population served by WWTPs 1 and 2 (according to the data in the national database of the Robert-Koch-Institut, Berlin, Germany; https://www.rki.de/DE/Content/InfAZ/N/Neuartiges_Coronavirus/Daten/Fallzahlen_Kum_Tab.html).

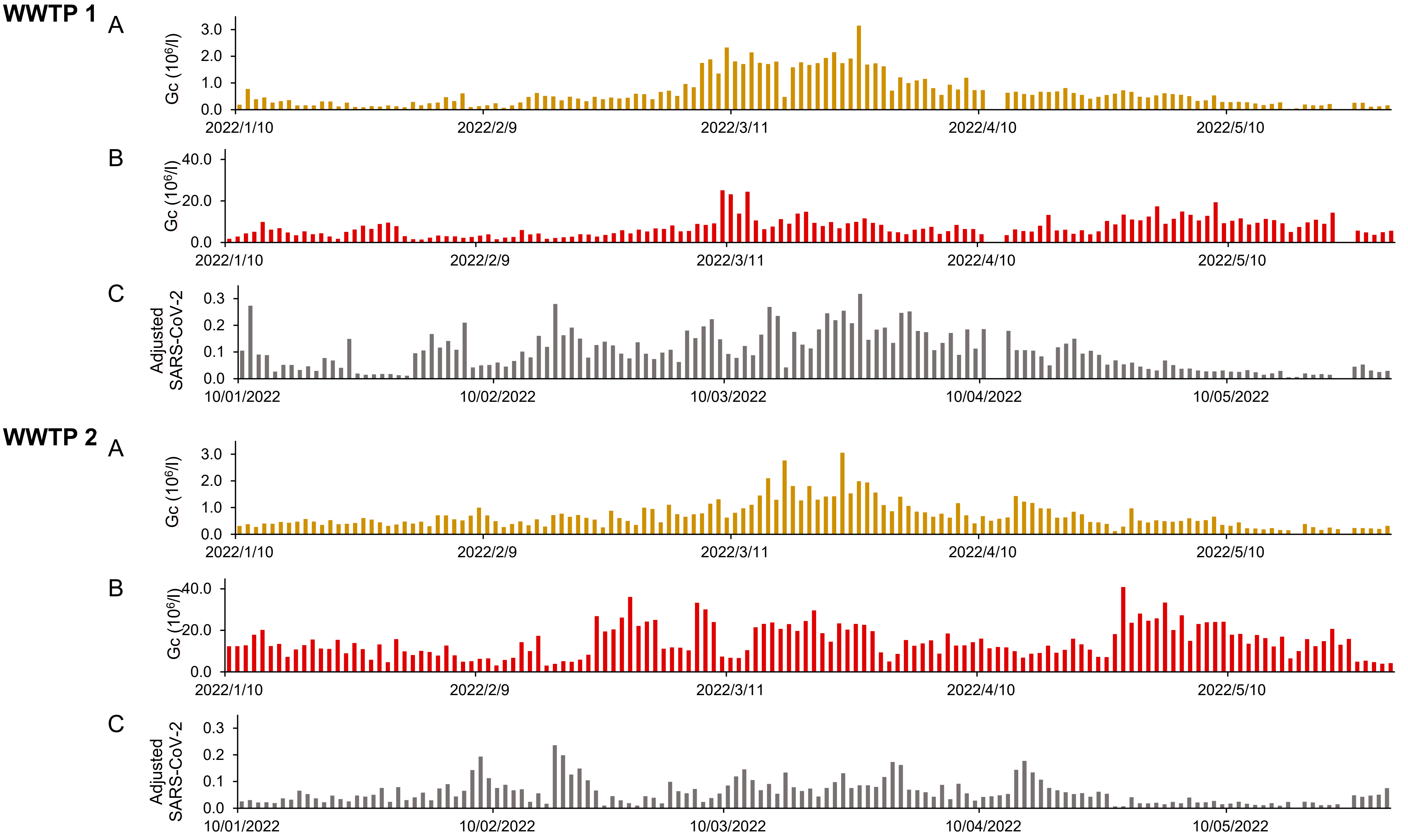

Figure S2. Concentration of SARS-CoV-2 (E gene; A), of crAssphage (B) and crAssphage-adjusted concentrations of SARS-CoV-2 (SARS-CoV-2 E gene gc / crAssphage gc; C) in wastewater of WWTPs 1 and 2.

Figure S3. Standard curves of real-time PCR for detection of influenza virus A and B (Influenza Screen & Type RT-PCR kit 4.0; Altona Diagnostics) after testing 1:4 dilutions (5 parallels per dilution) of standard (AccuPlex Flu A/B and RSV verification kit; LGC Seracare).

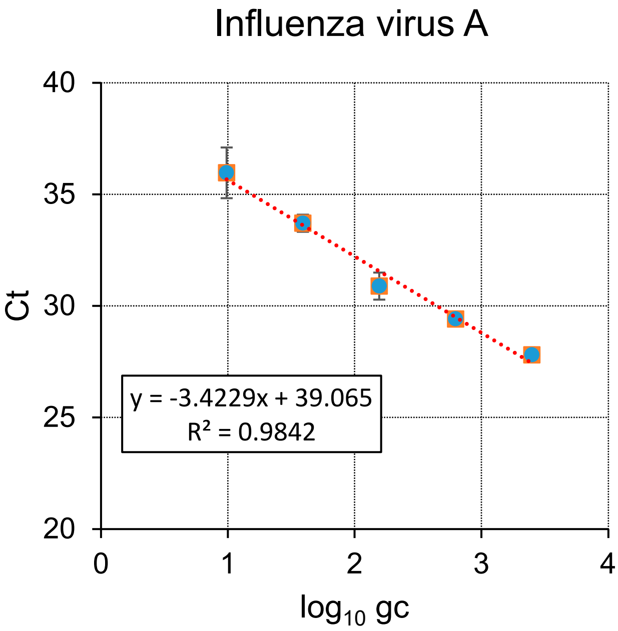

PCR efficiency: 96,0%

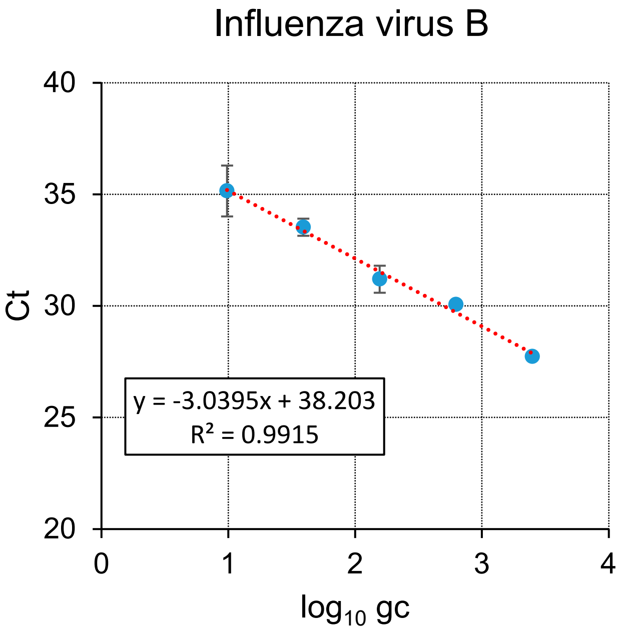

PCR efficiency: 113,3%
